# A Singular Case of Pregnancy at the Full Period, with Early Death of the Fœtus

**Published:** 1850-07

**Authors:** T. C. Osborne

**Affiliations:** Erie, Alabama


Art. II.—*A Singular Case of Pregnancy at the Full Period, with Early Death of the Fœtus.* By T. C. Osborne, M. D., of Erie, Alabama.

Mrs. ——, is twenty-three years of age, habitually healthy, with florid complexion and decided en bon point; married five and a half years; and has three healthy children. The fourth conception dates from the latter part of July, 1849, and up to the 20th of October, every evidence usual to the period was of a character unmistakeable and palpably impressive, but onward to the ninth month, all signs of pregnancy disappeared, and in their stead trains of non-descriptive sensations, despondency and painful misgivings of some approaching calamity, were present almost continually.

On the 6th of January, 1850, a slight stain of sero-sanguineolent fluid issued from the vagina, accompanied with a few feeble contractions of the uterus, which all passed off in a few hours, without affording any alleviation to the mental or physical distress.

At the expiration of the ninth month, on the 22d of April, labor commenced, and after twelve or fourteen hours extraordinary suffering, an entire ovum of three months development was forcibly expelled.

Upon rupturing the membranes, three ounces of thick, turbid liquor escaped, and a rather muddy, shrivelled embryo, five inches in length, was discovered, attached by a bloodless chord ten inches long, to a completely cartilaginous placenta of two and a half inches diameter, its uterine surface granular, florid, and unyielding to pressure throughout, while the fetal surface exhibited a confused plat of enlarged, tortuous and turgid vessels enclosing blood that was black and firmly coagulated. The mouth, ears, and extremities of the embryo were proportionally large, but the gender could not be distinguished by any sexual development.

On page 98, vol. v, 2d series of this Journal, it will be seen I have reported a case similar in many respects to the foregoing, and the analogy will be more perfectly seen, by inserting here that the residences of the subjects were adjacent; their temperament and contour very nearly alike, and the tendency to corpulency about the same. There are, however, several points of difference in the cases, of a very interesting nature, in respect to the ovum. In the first case the embryo was nearer maturity when it ceased to live; was not retained so long after its death; was in a state of incipient putrefaction, and the pathological anatomy of the placenta, although having the same morbid action, was of a different general feature. There were fissures corresponding to the depth of the disease, different degrees of cartilaginous formation, and an apoplectic clot on the uterine surface of the first, while a striking uniformity of unbending cartilage existed in the second, with a total absence of a trace of fissure.

Again, the fetal surface of the former presented little or nothing abnormal, while the latter seemed to have suffered intensely from apparent strangulation. If two cases of the kind, so nearly similar, has ever occurred to any other observer, it has not been my fortune to hear of them.

Erie, Alabama, May 4, 1850.

